# A Practical Guide to Developing a Research Communication Strategy for Low Income and Middle-Income Countries

**DOI:** 10.1192/bjo.2024.297

**Published:** 2024-08-01

**Authors:** Bushra Ali Shah, Nadeem Gire, Syed Kashif, Salman Farooqui, Mohamad Khalifa Fofana

**Affiliations:** 1 Pakistan Institute of Living and Learning, Karachi, Pakistan; 2 University of Bolton, Manchester, United Kingdom; 3 Global Mental Health Cultural Psychiatry Group, Manchester, United Kingdom; 4 University of Manchester, Manchester, United Kingdom

## Abstract

**Aims:**

Despite increasing research activities in low- and middle-income countries (LMICs), the impact of research is challenging to measure and assess, given the myriad of systemic challenges that exist in these settings. Socio-political constraints, limited education prospects, cultural stigma, restricted access to training and development, and the poor research infrastructure in low-resource settings contribute to the widening gap between evidence and policy, and in turn, creates serious barriers to mental health care in these countries. One of the main barriers to the effective implementation of research in LMIC settings is poor governance and dissemination. Given the lack of standard guidelines, there is an unmet need to develop a communication framework that will strengthen the implementation of evidence-based findings in policy and practice. As a first step towards this goal, our aim was to develop a research communication strategy to enhance research outcomes in LMICs.

**Methods:**

We conducted a narrative synthesis to understand the key factors which may be used to measure both the reach and depth of research impact and communication within LMIC settings.

**Results:**

Our analysis outlined metrics and indicators of research impact including academic outputs, social media insights, capacity building, Patient, Public Involvement & Engagement, policy development, collaboration and partnership, and health and economic benefits. Based on our findings, we formulated steps to support the development of a research communication strategy which has the potential to guide an effective research impact framework and ultimately help bridge the evidence-treatment gap in LMICs. 1) Identify stakeholder groups, 2) Employ Theory of Change approaches and community engagement, 3) Explore channels of communication, 4) Developing a ‘Plain English’ summary, 5) Incorporating cultural and contextual factors, 6) Leverage digital technology and social media.

**Conclusion:**

Participatory approaches to research communications are of paramount importance in informing and implementing evidence-based findings in low-resource settings. Research communication is a prerequisite to the development of an effective impact assessment framework that supports the prioritisation of key areas of public mental health in low-resource settings. Developing a comprehensive communication strategy which leverages culturally appropriate communication strategies targeted at diverse stakeholder groups, may amplify research impact, under a holistic framework which prioritises the delivery of evidence-based mental health care in LMICs.

